# Chimpanzees engage in competitive altruism in a triadic ultimatum game

**DOI:** 10.1038/s41598-024-53973-6

**Published:** 2024-02-09

**Authors:** Alejandro Sánchez-Amaro, Luke Maurits, Daniel B. M. Haun

**Affiliations:** 1 Department of Comparative Cultural Psychology, Max Planck Institute for Evolutionary Anthropology, Leipzig, Germany; 2 Department of Psychology, Faculty of Natural Sciences, University of Stirling, Stirling, UK; 3 Leipzig Research Center for Early Child Development, Leipzig University, Leipzig, Germany; 4 LeipzigLab, Leipzig University, Leipzig, Germany

**Keywords:** Chimpanzees, Competitive altruism, Ultimatum game, Strategic decision-making, Triads, Animal behaviour, Social evolution

## Abstract

Partner choice promotes competition among individuals to be selected as a cooperative partner, a phenomenon referred to as competitive altruism. We explored whether chimpanzees engage in *competitive altruism* in a triadic Ultimatum Game where two proposers can send offers simultaneously or consecutively to a responder who can only accept one of the two competing offers. In a dyadic control condition only one proposer at a time could send an offer to the responder. Chimpanzees increased their offers across trials in the competitive triadic, *but not* in the dyadic control condition. Chimpanzees also increased their offers after being rejected in previous triadic trials. Furthermore, we found that chimpanzees, under specific conditions, outcompete first proposers in triadic consecutive trials before the responder could choose which offer to accept by offering more than what is expected if they acted randomly or simply offered the smallest possible amount. These results suggest that *competitive altruism* in chimpanzees did not emerge just as a by-product of them trying to increase over previous losses. Chimpanzees might consider how others’ interactions affect their outcomes and engage in strategies to maximize their chances of being selected as cooperative partners.

## Introduction

Partner choice dynamics are fundamental for understanding animal sociality. They are crucial for explaining how animals, including humans, trade commodities reminiscent of market transactions^1^. These dynamics can lead to competition among individuals to be selected as cooperative partners, often referred to as competitive altruism^2,3^, competitive helping^4–6^, or reputation-based partner choice^7,8^. According to Hardy and Van Vuigt, competitive altruism is the "process through which individuals attempt to outcompete each other in terms of generosity"^3^. Competitive altruism has played a significant role in the evolution of human cooperation and morality^2,9,10^. By being generous, individuals can increase their reputation as good cooperators. For instance, increases in reputation or status would yield individuals benefits through access to mutual cooperation or indirect reciprocity^11^.

Several studies have explored whether humans engage in competitive altruism to increase long-term benefits. In a study by Barclay and Willer^12^, pairs of adults participated in a game where they could simultaneously donate any number of dollars (from 0 to 10). The donations were then doubled and equally distributed between the two participants. Afterward, a third player who did not participate in the game was paired with one of the two previous players. In two study conditions, the pairing occurred randomly, and the new player was either informed or not about the other players' previous donations. In a third condition, the new player was informed about the earlier contributions and could directly choose with whom to play the same game again. As a result, participants donated more when they knew that the third player could choose the best cooperative partner to play a new game, but not in the first two conditions. Following a similar design, Herrmann and colleagues^13^ found that 8yo (but not 5yo) children shared more when they were aware of potentially being selected to play again later. Other studies have demonstrated competitive altruism/helping dynamics when participants’ contributions could directly benefit those individuals choosing. For instance, Raihani and Smith^4^ found that men were more likely to donate to attractive women fundraisers after other men had donated before them. When the fundraisers were men or less attractive women, such competitive altruism dynamics did not arise. Here, men did not just increase their reputation as good donors, but also directly helped specific fundraisers.

Whether people engage in competitive altruism has also been studied using game theory models such as the Ultimatum Game (UG). The UG portrays a situation where a proposer can divide a finite amount of resources between herself and the partner. The partner can choose whether to accept or reject the division altogether so that both participants obtain zero rewards. Using this paradigm, Chiang^14^ showed that when players are aware of others' previous offers and acceptance decisions in previous UGs and are allowed to choose with whom they would prefer to interact in further rounds of the game, players' offers increase compared to conditions in which individuals are randomly paired with each other. Specifically, prosocial proposers and tolerant responders tend to select each other for mutual benefit while less cooperative proposers need to adapt and behave more generously after being paired with less tolerant responders. Even more relevant for our study, Roth and colleagues^15^ found that when one responder could only accept one of several proposers' offers, competition arose among proposers who rapidly increased their prosocial offers favoring the responder (see^16^ for similar results in a Competitive Altruism condition using the Dictator Game).

Besides humans, other animals may also engage in competitive altruistic dynamics during social interactions. Through partner choice and commodity trades, individuals may compete between them to become more attractive social partners. For example, observational studies have found that many primate species prefer to groom higher-ranking individuals from whom they can obtain agonistic support against other group members^17^.

Experimental studies on captive great apes suggest behavioral preconditions that could facilitate the emergence of competitive altruism. For instance, chimpanzees can select the best of two cooperators based on a history of previous interactions^18^ and can distinguish between friends and non-friends before risking access to food rewards in a trust game^19^. However, perhaps the most relevant example comes from a study where pairs of chimpanzees and bonobos were presented with an UG in which the responder could access alternative rewards besides the proposers' offer^20^. The alternative could be used as leverage for the responder and, in turn, influence the proposers' offer. The authors found that apes were sensitive toward the responders' alternatives and proposed fairer offers. However, in these previous studies, apes were not competing to be selected as social partners. Thus, whether non-human animals engage in competitive altruistic dynamics to reap benefits from cooperative interactions remains an open question.

In our current study, we present triads of chimpanzees with a modified repeated UG where two proposers can make their offers consecutively or simultaneously, and a responder can only accept one of the two offers. Human studies with multi-person UGs show that people compete for their bids to be accepted^15,21^, fulfilling the conditions for competitive altruism to emerge by offering more than their partner, with the caveat that in multi-proposer UGs proposers compete to offer the most to the responder, the responder in turn directly benefits from such interaction and one of the proposers gain direct short-term benefits. In contrast, in more common competitive altruism tasks^12,13^, individuals compete with each other to be selected by third-party members to engage in future interactions and gain long-term reputational benefits (although see^4^ for a scenario in which individuals increase their reputation by directly benefitting the potential third-party). Our task could also be interpreted as an economic bargaining game in which the proposers try to obtain the benefit from the responder by offering more than their partner (after all, the apes are playing the Ultimatum Game^22^). In any case, if competition between proposers occur as we expect, the result is an increase in generous offers towards the responder, which is the key aspect we aim to investigate.

In our experimental setting, each proposer can send any number of grapes from 1 to 8 to the responder. Our approach differs from previous UGs in great apes, where apes can choose among preselected food constellations^20,23–25^ or tokens representing different offers^26^. High offers can be costly for the proposer in the short term since it automatically lowers the maximum income she receives. For example, if the proposer offers six grapes, she can only obtain the two grapes left on her tray if her offer is accepted by the responder. However, individuals who consistently send high offers may be more likely to be accepted and benefit over time. Eventually, if both proposers want to benefit, they should outcompete each other in generosity.

Furthermore, we compare these repeated triadic situations to repeated dyadic scenarios in which only one proposer has the opportunity to make an offer. Previous dyadic work with chimpanzees found that proposers usually offer the minimum while responders accepted any non-zero offers^16–18^ but see^26^. Therefore, based on previous UG studies with great apes, we predicted that the total sum of offers would be higher in triadic compared to dyadic scenarios as in the latter their offers would be less likely to be rejected (see https://osf.io/9m35c). Additionally, we expected chimpanzees to increase offers across trials in the triadic compared to the dyadic scenario assuming that the proposers and the responder would prefer to improve their benefits^20,23–26^. To evaluate this hypothesis, we modeled the probability of offering more in the final compared to the initial trials within one session and whether these differences between final and initial offers increased over sessions. We also investigated under which conditions apes increased their offers relative to previous trials to shed light on the mechanisms explaining competitive altruism in chimpanzees. We expect chimpanzees to increase their offers if their previous offer was rejected.

Finally, we investigated whether and when chimpanzees would behave strategically in consecutive trials, when second proposers had the opportunity to make an offer after seeing the first proposer’s offer but before the responder made a choice. If offering behavior is mainly influenced by the proposer’s previous gains and losses, second proposers would be more likely to increase their offers (and to outbid a first proposer) after being rejected on the previous trial. If instead they focus on the current first offer from the opposite proposer rather than on the result of the previous trial, apes should attempt to outcompete their partners' offer regardless of whether they benefit or not in the previous trial. We predicted that those playing second would have a clear incentive to outbid the initial offer. Crucially, not all trials offer the same motivation to outbid their partners, e.g. an initial offer of 1 grape can be outbid with an offer of 2 or 3 grapes, still securing 5 or 6 grapes for the proposer, while outbidding an initial offer of 6 grapes results in a reward for the proposer of at most 1 grape. For that reason, we evaluated whether the likelihood of chimpanzees outbidding their competitor varied depending on the value of the first offer. We expected chimpanzees to outbid first offers when the resulting reward would be equal to or higher than the reward offered to responders.

## Results

In our first pre-registered model (https://osf.io/9m35c) we assessed whether apes offered different quantities of food between dyadic and triadic conditions. We found that chimpanzees offered similar amounts of food in dyadic and triadic conditions (see Fig. S1 in the ESM). The posterior predictive distribution for the total proportional offer in the dyadic condition had a mean of 0.37 with a 95% HPD interval of [0.01, 0.90]. For the triadic condition, the posterior predictive mean was slightly lower at 0.31 with a 95% high posterior density (HPD) interval of [0.02, 0.75]. Roughly, this means that by session 9 they were offering an average of 3 grapes in dyadic trials and of 2.5 grapes in triadic trials. The 95% HPD interval for the difference in total proportional offer between the two conditions did not exclude zero [− 0.66, 0.53] and had approximately equal probability mass on either side of zero. This suggests that any systematic difference in total offering behavior between the dyadic and triadic conditions is minimal relative to the variation in offers within both categories. Likewise, in our second pre-registered model we found that chimpanzees offered similar food amounts in consecutive and simultaneous triadic trials. The posterior predictive distribution of total proportional offers in the consecutive triadic condition had a mean of 0.31 with a 95% HPD interval of [0.01, 0.83]. For the simultaneous triadic condition, the posterior predictive mean was slightly lower at 0.25 with a 95% HPD interval of [0, 0.76]. On average, half-way through the study apes were offering around 2.5 grapes in consecutive triadic trials and 2 grapes in simultaneous triadic trials. While the posterior probability that the mean offers in consecutive trials exceeded that in simultaneous trials was 0.82, the 95% posterior predictive interval for the difference in offer between conditions [− 0.56, 0.7] makes it clear that any tendency to make higher offers in the consecutive condition is small relative to the variation in offers in both conditions. See Fig. S2 in the ESM and more details in Sect.  S1 of the model summaries file (MSF).

**Figure 1:**
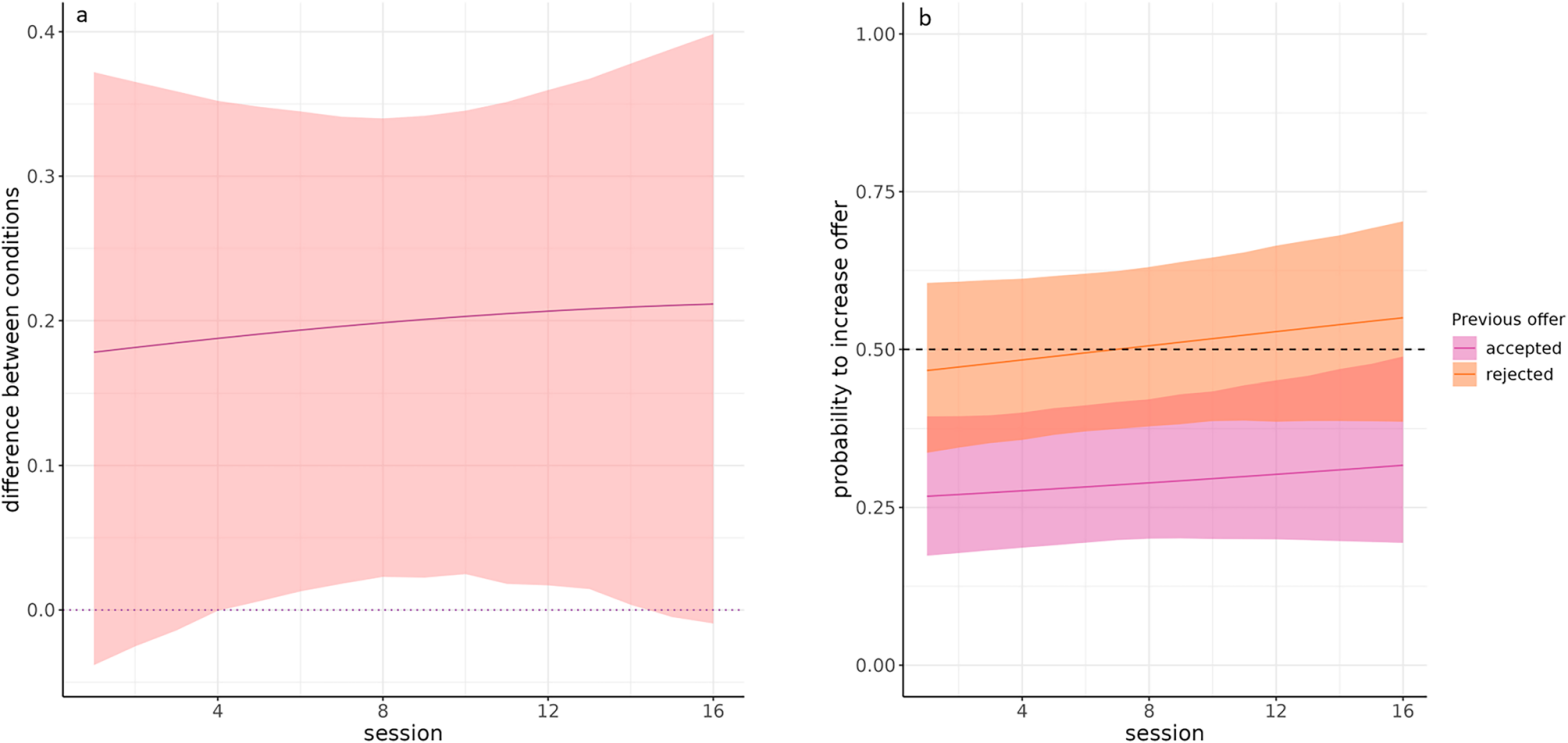
(**A**) Plot representing the difference in probability of offering more in the last trial than in the first trial between dyadic and triadic conditions (posterior mean plus 95% HPD); (**B**) plot representing the different probabilities of a triadic proposer offering more than they did in the previous trial based on whether the previous offer was accepted or rejected (posterior mean plus 95% HPD).

However, apes could have employed different offering strategies in dyadic and triadic sessions at the trial by trial level, despite offering similar total amounts. To answer this question, we focused on the apes' tendency to increase their offers over the course of a given session. We found that chimpanzees' overall tendency to offer more in their final trial compared to their initial trial was higher for triadic sessions than for dyadic sessions. Marginalizing over all sessions we estimated that the probability of a proposer offering more in their final trial of a dyadic session compared to the first trial of that session was 0.37 with 95% HPD interval [0.23, 0.53]. Across sessions, the posterior mean estimate of this probability increased from 0.34 to 0.41. In contrast, in the triadic condition, the mean probability marginalizing across sessions was 0.57 with 95% HPD interval was [0.39, 0.73]. This probability increased across sessions from 0.52 to 0.63. Notably, for 12 of the 16 sessions, the 95% HPD intervals for the *difference* in apes' probabilities to offer more in the last vs. the first trials of a session between dyadic and triadic sessions excluded zero (see Fig. 1a). Variation in behavior across triads was relatively limited. While the extent of the dyadic vs triadic contrast was stronger or weaker in some triads than others, all the per-triad posterior probabilities that increasing offers were more probable in triadic sessions than dyadic sessions were above 0.9 (see Fig. S3 in the Supplementary Materials (ESM), and more model details in Sect. S2 of the MSF). In short, after gaining some experience with the apparatus, apes behaved differently between the dyadic condition, wherein they were more likely to *decrease* their offers within a session, and the triadic condition, wherein they were more likely to *increase* their offers within a session. This finding is consistent with the previous finding that there is no appreciable difference in the *total* offer between conditions if the first offers in dyadic sessions are higher than the first offers in triadic sessions, and indeed this was the case (mean first dyadic offer 3.52, mean first triadic offer 2.27).

In order to investigate the mechanisms underlying the increases of offers in the triadic condition demonstrated above, we assessed whether apes were more likely to increase their offers after rejections in the triadic UG than in trials in which their previous offer was accepted. We found that apes were less likely to increase their offers relative to their previous offers after that offer was accepted (posterior mean probability of 0.29 with 95% HDP interval [0.19, 0.42], entirely below chance) than when that offer was rejected (posterior mean probability of 0.51 with 95% HPD interval [0.36, 0.64]). This tendency varied minimally across the various combinations of proposers and responders, and in every case the posterior probability that increasing offers were more common after rejections than after acceptances was 1. Estimating these probabilities separately for simultaneous and consecutive triadic trials revealed only minor differences (see Fig. S4 in the ESM and more model details in Sect. S3 of the MSF). Our results suggested that apes reacted rationally to the previous rejections by increasing their offer on the subsequent trial during triadic situations (see Fig. 1b). However, even though apes tended to increase their offer relative to their own previous offers after experiencing rejections, they did *not* tend to match or increase their offers over the value of the last winning offer when their offer was rejected. Apes probability to match or increase over the winning offer when it was their own previous offer was 0.45 (95% HPD interval [0.28, 0.63]) and apes' probability to match or increase over the winner offer when it was not their previous offer was 0.36 (95% HPD interval [0.21, 0.54]). Chimpanzees reacted to their offers being rejected but not to the value of the accepted offer the other proposer sent in the previous trial. Such a reactive strategy could trigger competitive helping on the surface, despite the possibility that apes did not really compete against other proposers but simply increased their offers given that the previous ones did not work out. To assess whether apes tried to outcompete others proposers we thus focused on consecutive trials. Specifically, we assessed whether second proposers would try to actively outcompete first proposers offers before the responder could decide.

We found that while chimpanzees offering as second proposers became more likely to "outbid" first proposers across sessions (posterior probability of positive session slope 0.76), the 95% HPD interval for this probability never excluded chance (posterior mean and 95% HPD interval for final session: 0.45, [0.25, 0.67]). However, when we estimated separate outbidding probabilities for each possible first offer and considered the outcome of the second proposer’s previous offer, a more nuanced picture appeared. The probability of second proposers, who had their offer rejected in the previous trial, outbidding first proposers increased with sessions (posterior probability of positive slope 0.72), while for previously accepted proposers there was no change across sessions (posterior probability of positive slope 0.45). By the final session, evidence was moderate to strong that previously rejected proposers were outbidding initial offers of 0 to 2 grapes at above chance levels (posterior probability 1, 0.97, 0.79, respectively) and initial offers of 5 or more grapes at below chance levels (posterior probabilities 0.08 or below). Meanwhile, in the final session previously accepted proposers only showed clear evidence of outbidding initial offers of 0 or 1 grape at rates above chance (posterior probability 0.99 and 0.92, respectively).

The fact that after having a previous offer rejected, second proposers tend to only outbid initial offers of less than 4 grapes is consistent with our prediction on the basis of proposers being motivated by a desire to end up with more grapes than the responder. However, the same pattern could arise simply from a bias toward making low offers: initial offers of 1 grape will be frequently outbid by a second proposer who simply offers a random low number of grapes without considering the first offer, while higher initial offers will not. To assess whether our data can be explained in this way, we estimated “baseline" outbidding probabilities by simulating a large number of consecutive sessions where both first and second offers were sampled independently from a fixed distribution based on first offers made in actual consecutive sessions. From these simulated sessions, we computed the proportion of trials with each possible first offer where the second offer was higher. The fixed distribution of simulated offers was based on only observed first offers because second offers do not necessarily reflect the proposer's preference for low offers over high offers –second offers may be chosen to outcompete the first offer. Even first offers are not necessarily “pure” indicators of offering preference in the absence of competitive or strategic behavior, as first offers late in a session happen in the context of preceding trials. However, using only first offers from the first trials of each session would result in too few datapoints to reliably estimate preferences. Therefore, we estimated a distribution of preferred offers using all observed first offers, but weighting trials early in a session higher than trials late in a session, and weighting early sessions higher than late sessions (see the model summaries file for full details). In simpler terms, as a baseline we considered how likely a first offer would outcompete another first offer by chance.

**Figure 2:**
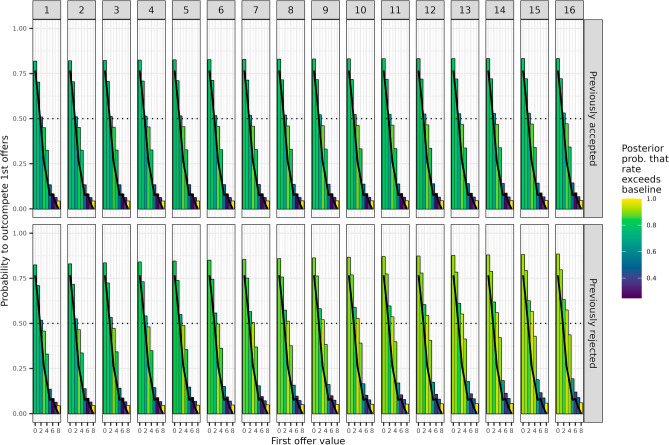
Plot representing the posterior mean probabilities of a second proposer to outbid a first proposer after the second proposer was previously accepted (top row) or was previously rejected (bottom row). The probabilities to outcompete first proposers are compared to the baseline outbidding probabilities estimated from first offers only (blue) and to chance levels (dotted line) across the 16 test sessions. The colour gradient represents the strength of evidence that the observed rate of outbidding exceeds the baseline.

Comparing our model’s predictions against a reference point of outbidding at rates higher or lower than the baseline expected from a simple preference to offer lower numbers of grapes more often than higher numbers, reinforced the interpretation in terms of strategic behavior and motivation (Fig. 2). By the final session, previously rejected proposers were outbidding initial offers of 0 through 4 grapes inclusive at rates above the baseline (posterior probabilities 0.86 or higher). Even previously accepted proposers appeared to be outbidding initial offers of 3 or 4 grapes at rates above the baseline (posterior probabilities of 0.87 and 0.79 respectively). Furthermore, under the baseline initial offers of 5 grapes were only slightly less likely to be outbid than initial offers of 4 grapes (probabilities of 0.17 and 0.08 for 4 and 5 grapes), while the model’s posterior mean outbidding probabilities drop sharply when crossing this motivational threshold (0.44 to 0.19 for previously rejected proposers, 0.34 to 0.14 for previously accepted). Random effects indicated more variation across proposers and responders in the outbidding behavior than other reported behaviors, but nevertheless the posterior probabilities of outbidding initial offers of 3 or 4 grapes at above baseline rates exceeded 0.8 for 9 out of 15 pairs of second proposer and responder, and only a single one of the five second proposer never exceeded 0.8 across all responders. Two second proposers showed modest evidence (posterior probability 0.77 or higher) of outbidding initial offers of 4 grapes at higher than baseline rates against multiple responders (see Fig. S5 in the ESM and more model details in Sect. S4 of the MSF).

## Discussion

While chimpanzees offered similar total shares to responders in the triadic condition of our UG compared to a dyadic control condition overall, the probability of offering more during the last trials, compared to the first trials of a session, increased over time in the triadic condition. In contrast, in the dyadic condition, the probability to offer more in the last compared to the first trials remained clearly below chance until the latest sessions. Significantly, for the great majority of sessions, the probabilities of offering more in the last compared to the first trials were clearly distinguishable between the two conditions. These results are consistent with the idea that apes engaged in competitive altruism when two proposers had to compete to get their offers accepted.

One way to explain how competitive altruism emerged across our study is by looking at apes' reactions to their rejected offers in triadic trials. We found that apes were sensitive to the rejection of their offers, being clearly more likely to increase their subsequent offers after a rejection than after an acceptance (somewhat similar to the strategy “reluctantly increasing proposer” that we used for our power analysis, see ESM S1). Nonetheless, the probability of offering equal or more than in the previous trial when their last offer was accepted increased slightly over sessions, perhaps as a strategy to keep being accepted. However, when the last offer accepted came from the opposite proposer, they did not tend to match it or offer more in the subsequent trial. In our opinion, this pattern of results is consistent with the idea that chimpanzees focused their attention primarily on what they were proposing and maybe did not keep in mind the offer made by the opponent in the previous trial.

Our results thus far suggest that, as in previous social settings, chimpanzees reacted to aversive scenarios in ways that helped them maximize their probability to benefit in the future^18,27–30^. Specifically, we argue that chimpanzees reacted flexibly to rejections by remembering and outbidding what they had previously offered^31,32^. This strategy increased their chances of being accepted in subsequent trials—even though that meant giving up more rewards. In consequence, competitive helping possibly arose simply as a by-product of both proposers being either accepted or rejected in any given trial as responders consistently accepted the highest offer most times^2^. Following the strategies we used in our power analysis (see ESM S1), responders behaved rational—“the more for me, the better”. In line with this argument, we found that chimpanzees who had been previously rejected were more motivated to outcompete first offers during consecutive trials. They increased their offers over sessions to the point that they outbid first proposers above chance levels when those initial offers constituted half or less than the total rewards. Nonetheless, this pattern did not simply emerge due to a preference for making low offers. They consistently offered over the baseline of outbidding probabilities based on first proposers’ offers for low and mid-value rewards. Even more interesting, chimpanzees also appeared to outbid initial offers of three and four grapes above the baseline after they had been previously accepted. In other words, even though apes were less motivated to outcompete offers after being previously accepted it does not mean they did not try to outbid their partners in some occasions. The result is that when initial offers were low, chimpanzees outcompete first proposers over chance during last test sessions but, in general, their likelihood to outcompete first offers did not differ from the baseline of outbidding probabilities. However, as the likelihood to outbid higher offers was harder to explain by a simple preference to offer less, apes’ tendency to outbid initial offers of three and four over the baseline of outbidding probabilities arose, suggesting that under favorable circumstances chimpanzees’ strategies contributed to the emergence of competitive altruism during consecutive triadic trials.

Most importantly, these results hint at the possibility that apes did not just react to their offers being previously accepted or rejected. Instead, in line with previous work on the UG, we provide evidence that chimpanzees can focus on the standing offers made by the first proposer, reacting to what the opponent proposer was offering while anticipating what the responder could accept. Our results extend previous work on apes' theory of mind abilities^33–36^ to show that chimpanzees can strategize before others make decisions in social dilemmas, possibly inferring what others prefer^35^.

However, apes did not always use optimal strategies to get their offers accepted and were primarily influenced by the outcomes of the previous interactions. We believe that apes did not show more strategic behavior because, as reported in previous social dilemmas^37,38^, they may have faced a constant trade-off between minimizing offers to obtain higher benefits and offering more than the partner to be accepted. In fact, chimpanzees were mainly motivated to outbid low offers, especially when their offers had been previously rejected. In contrast, apes showed a marked reluctance not just to outbid but initially propose large offers, primarily when those constituted more than half of the initial outcome, maybe because they would automatically obtain less than their partner.

This reluctance to outbid high offers, attributable to predicted benefits not being worth the high investments, may act as a "brake" on the tendency of competitive altruism to produce ever increasing offers, explaining why the total offers in triadic sessions failed to exceed those in dyadic sessions regularly. An alternative explanation for such reluctance and lack of consistent strategic behavior is that apes might have found challenging to observe and count how many grapes were necessary to outcompete the partner when both offers were high. However, whereas apes indeed have difficulty correctly choosing the highest allocation when the ratios between two quantities get closer to one^39^, we also found that responders accepted the highest offer in 88% of the triadic trials, and when they failed the average ratio between the two offers was 0.46—a value within the range of ratios where apes usually select the larger quantity out of two. Relatedly, apes' stick use and overall visibility could have also hindered their focus on others' offers, especially during simultaneous trials. Previous work on trap tasks shows that apes using tools to solve mazes had a more challenging time avoiding traps than apes who could use their fingers to move rewards through the maze^42,43^. It is thus possible that apes mainly monitored the food they sent with the stick.

It is also worth mentioning that chimpanzees usually offered responders more food than expected during dyadic trials. Unexpectedly, proposers did not act rationally in this situation. This pattern of results is especially relevant relative to previous studies suggesting that apes behave as rational maximizers in dyadic UGs^23–25^. If apes had consistently tried to maximize their rewards in dyadic trials, they should have offered only one reward instead of three. Therefore, our results tend to align more with studies such as the one conducted by Proctor and colleagues^26^, where they found that chimpanzees, only as proposers though, did not always offer the rewards that benefitted them the most. Nonetheless, there are significant differences between^26^ and our study. In their study, chimpanzee proposers transferred tokens of different values to the responder, and the latter could decide whether to accept or reject the offer by returning the token to the experimenter or keeping it. In our case, the experimenter did not play such an active role during the trial, and the proposers directly offered food to the responders. Furthermore, as highlighted by other researchers, chimpanzees were not trained with the refusal option in that study^44^. In contrast, in our study chimpanzees were trained to refuse options as responders and were also aware of the consequences of refusing those options.

Thus, it is unclear whether our result in dyadic trials arose because the setting facilitated apes choosing the quantity they wanted to offer—a feature necessary for competitive altruism to emerge in the triadic condition. Another possibility is that the same design that facilitated the emergence of competitive altruism in triadic trials also allowed "mistakes" during dyadic trials resulting in proposers offering more than expected. Support for this argument is that responders maximized their choices when they faced a dichotomous choice between two final offers. These choices were similar to the ones presented to chimpanzees in previous UGs. In all, the complexity and the freedom of choice could help explain why chimpanzee proposers did not behave as rational maximizers in our dyadic version of the UG.

Finally, we would also like to highlight that, despite the small number of chimpanzees that participated in the last stage of the study, our results accurately represent the behavior of the study participants. Across a variety of models, triads and proposers’ behavioral patterns are consistent with the reported population level trends. However, we remain agnostic whether the same patterns would replicate in another chimpanzee population consisting of other chimpanzees with different life histories or experience in cognitive tasks.

In conclusion, our results suggest that competitive altruism by giving more than the partner can emerge in chimpanzees, in our particular scenario, both as a by-product of the competition generated during the triadic trials and because apes used strategies aimed at out-competing others to get their offers accepted. In particular, our study builds on previous results by showing that apes are not only sensitive to the stable alternatives that other partners possess as leverage in social dilemmas^20,29^, but also to other competitors' possible offers and the value of these offers as alternatives for third-party individuals.

## Materials and methods

### Participants

Seven chimpanzees participated in the final test phase of the study (mean age = 25.58 years, standard deviation = 14.22 years, range = 37.41 years). In addition, six other chimpanzees participated in the first food quantity task but did not reach the criteria to advance to the training and test phases. See Table S1 in the supplementary electronic material (ESM) for more information about the participants. All chimpanzees lived in the Leipzig Zoo. They lived in large semi-natural indoor and outdoor enclosures, and the research was conducted in their sleeping rooms. Apes had a regular feeding schedule, daily enrichments, and water ad libitum during tests. During the study period, individuals were voluntarily separated from other group members. At any time, apes could stop participation and return to the indoor enclosure again.

The seven chimpanzees participating in the final test phase formed seven distinct triads. Five chimpanzees participated at least once as proposers and responders. The other two individuals only participated as responders after passing a quantity discrimination test (one could not achieve the criteria of the first training phase, and the other, for security reasons, did not participate in the test phase as a proposer).

### Ethics statement

The study was approved by an internal ethics committee from the Max Planck Institute for Evolutionary Anthropology and the Leipzig Zoo. Our work complies with the Weatherfall report 'The use of non-human primates in research'. The study also complies with the EAZA Minimum Standards for the Accommodation and Care of Animals in Zoos and Aquaria, the WAZA Ethical Guidelines for the Conduct of Research on Animals by Zoos and Aquariums, and the ASAB/ABS's Guidelines for the Treatment of Animals in Behavioural Research and Teaching. IAUCUC approval was not necessary to conduct this research.

### Materials

The Plexiglass apparatus sat on an open squared booth between three compartments. The apparatus had one accessible side per participant, forming an inverted U shape. The side of the responder rested on the interior frame of the booth. It consisted of two platforms (9 × 7 cm), one on the right and the other on the left side of the internal structure. A central sliding door inserted within the plexiglass panel impeded access to both platforms. On its initial position, the door automatically occluded two holes that gave access to the left and right platforms. The responder had to slide the door to either side to access one of the two platforms through one of the holes. When the responder accessed one platform, the other platform became automatically inaccessible since the door could not slide in the opposite direction anymore. Each platform was connected through a ramp with one of the proposers' sides. The two proposers' sides were perpendicular to the responder side, thus forming the inverted U shape. The proposers' sides were symmetrical and located in front of each other. Each side rested on the mesh separating the proposers' room and the central booth. Every side consisted of one rectangular tray (20 × 7 cm) that could flip approximately 30 degrees towards the proposer's side or the booth's interior. Both trays flipped in the same direction: when the left side tray flipped towards the mesh side, the right-side tray automatically flipped towards the interior side of the booth and vice versa.

**Figure 3:**
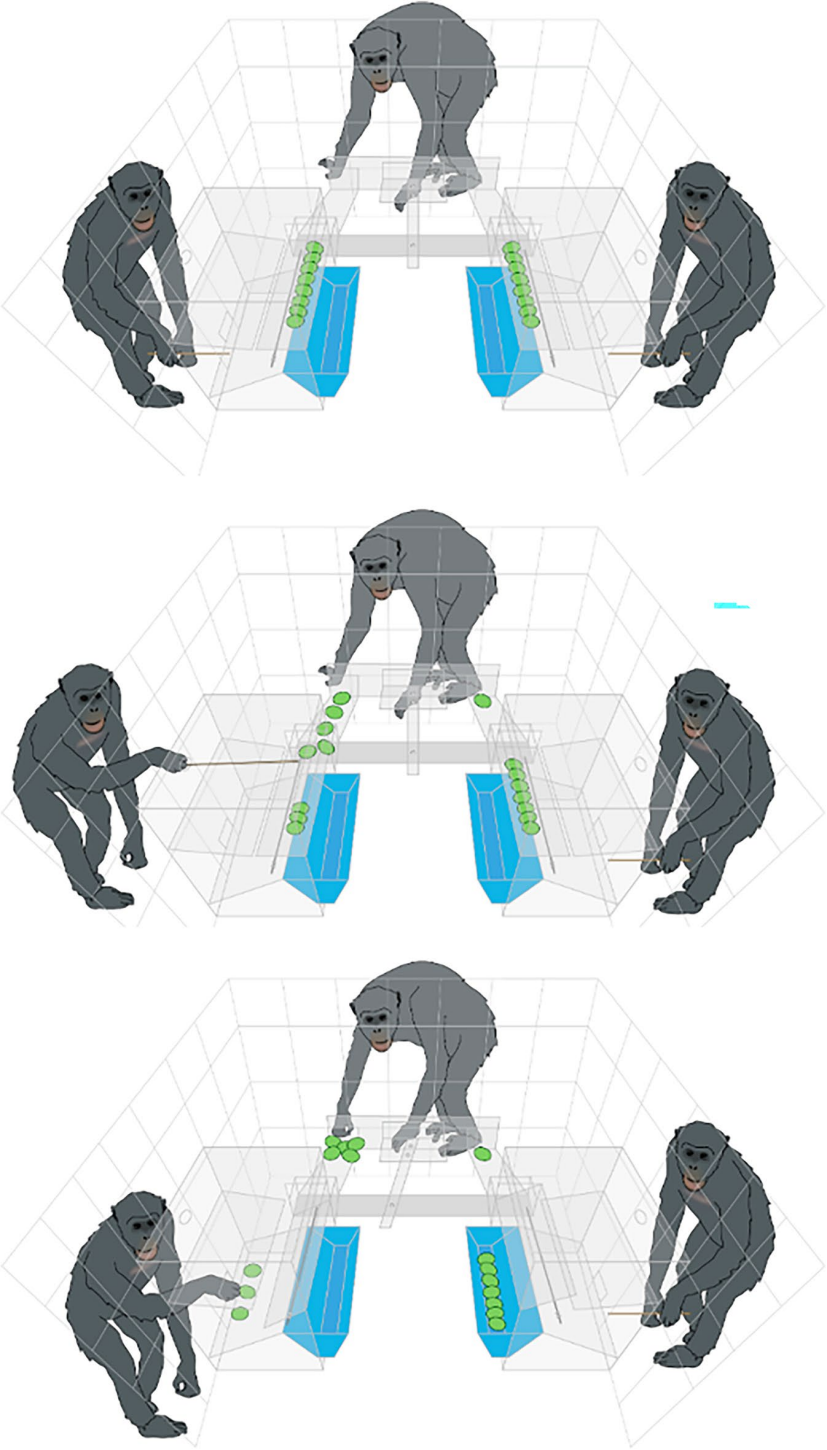
Representation of a consecutive triadic trial depicting the most relevant parts of the apparatus: in the top panel, proposers have not made their offer yet. In the middle panel, the left proposer is offering five grapes to the responder while the right proposer just offered one grape. The responder slides the door to her left to accept the five grapes and automatically rejects the single reward offer. In the bottom panel, the responder obtains the five rewards, and the accepted proposer obtains three rewards. The rejected proposer obtains zero rewards.

The grapes were located on top of the rectangular trays. Each proposer could use a wooden stick to push any number of grapes from 1 to 8 from the proposer to the responder's sides. Once the proposers made their offers, the experimenter locked their access to the apparatus. Simultaneously, the experimenter removed a peg blocking the responders' access to her side. When the device was unlocked for the responder, she could decide to access one of the platforms by sliding the central door right or left. The doors' movement connected with both rectangular trays and flipped them simultaneously, one towards the proposer whose offer was accepted and the other towards the booth's center. This way, the accepted proposer could obtain her share of the rewards. For instance, if the responder accessed the offer on the right platform, the proposer on the right side would automatically get her offer accepted and obtain a share of the rewards (eight grapes minus the number she offered) as her platform would flip towards her. In contrast, the proposer on the left would obtain no rewards as his platform would flip towards the booth's center, and all the rewards would fall into a central tray. See Fig. 3 for a graphical representation of a triadic test trial.

### Quantity discrimination test

The first task consisted of a quantity discrimination test in which individuals had to choose the highest of two quantities of food (i.e., a choice between two different amounts of grapes). Grapes were directly presented on the two platforms on the responder side. This way, apes learned how to access one of the two platforms to obtain the highest number of grapes while blocking access to the other. Furthermore, apes could not access the proposers' sides during the quantity discrimination test. On a given trial, the grapes could be presented simultaneously or consecutively. During simultaneous trials, the experimenter released one grape after another from his hands onto the platforms. With this procedure, the apes could observe how many grapes were placed on each platform. In the other half of the sessions, grapes were presented consecutively (half the time starting from the left side and half the time starting from the right side). We chose this procedure to better represent the nature of the offer presentation during future test sessions.

On every quantity discrimination session, chimpanzees were presented with eight consecutive trials. The number of grapes presented during test trials ranged from 1 to 8. Specifically, on each trial two different amounts were presented, only differing by one grape. Therefore, each session contained eight unique trial combinations (i.e., 1 vs. 2, 2 vs. 3 until 7 vs. 8 grapes). As we could not counterbalance the presentation side of each combination within a session—we would have needed 16 trials per session, we counterbalanced the presentation side between consecutive sessions. Individuals had to choose at least seven times the highest of the two quantities for two consecutive sessions to advance to the first training phase. The criterium mentioned above was difficult for some individuals, especially at high quantity counts (e.g., discrimination of 7 vs. 8 grapes). For that reason, we facilitated learning by introducing sessions in which chimpanzees could choose between quantities differing in three rewards (e.g., 4 vs. 7 grapes) with the addition of three motivational trials in which apes had to choose between 1, 2, or 3 rewards vs. none. Then, after they passed the test criteria (same as in the standard quantity discrimination test), they were introduced to sessions in which they could choose between quantities differing in two rewards (e.g., 3 vs. 5 grapes). Additionally, we included two motivational trials where apes could choose between 1 or 2 vs. 0 rewards. After passing the same test criteria, they returned to the typical quantity discrimination test. Therefore, depending on their performance, apes participated in 2–13 sessions until they passed the criteria, with five apes needing the extra sessions to facilitate their learning.

### First training phase

After the quantity discrimination test, six chimpanzees participated in the first training phase. In this phase, chimpanzees were tested individually and had access to all sides of the apparatus. Chimpanzees had to push all the grapes from the proposer trays to the platforms on the responder side, and there was a different food allocation on each. Apes could move freely between the three apparatus sides and push several times from each proposers' location before all the grapes fell on the responders' side. Nonetheless, chimpanzees usually pushed all the grapes from one proposer side before interacting from the other side. After moving all grapes, apes could only access one of the two offers from the responder side. They could never get rewards from the proposer side at this stage. Each session consisted of 6 trials in which apes had to choose between zero and four, two and four, or four and six rewards. Each pair of options was presented twice, and their presentation side was counterbalanced within sessions. Apes had to choose the highest of the two offers in at least 11 of 12 trials from two consecutive sessions. Five apes passed the training after participating in 2 to 9 sessions. A sixth ape struggled to use the sticks to send food to the responder. Therefore, she only participated as a responder during the final test phase. For security reasons, a seventh ape who had passed the quantity discrimination test did not participate in this training. Nevertheless, she still participated in the test phase as a responder.

### Second training phase

Five chimpanzees participated in the second training phase before the test phase began. In this training phase, apes again faced the same reward options and session structure as during the first training phase. The main difference is that in the second training phase, apes had no access to wooden sticks. That means they could not send offers from the proposer to the responder side. However, apes could still manipulate the apparatus from the responder side. By sliding the central door to the right or left, they could flip the flappable rectangular trays towards one side or another, thus accepting and rejecting the two offers at the same time. Since no grape was sent to the responder side, they had to move to one of the proposers' sides to obtain the accepted offer from that location. The rejected offer fell into the booth, out of the subjects' reach. We introduced this training to show them what occurred with the food that was not offered to the responder after acceptance and rejection from the responders' side. That is, we wanted them to focus on the movement of the flappable trays. All apes pass the training within 2 and 6 sessions.

### Test phase

Seven chimpanzees participated in the final test phase. Five apes participated in at least two triads, one as a proposer and the other as a responder. Another two apes participated only once as responders.

Each triad participated in 16 test sessions. Four triads started in the triadic condition, and three in the dyadic condition. Each session was composed of eight trials. Half of the sessions consisted of truly triadic interactions in which both proposers could make an offer to the responder, and the latter could only accept one of the two offers. Within every triadic session, apes could make offers simultaneously or consecutively. That means they had access to the grapes simultaneously or one after another. Each condition was presented in four successive trials. We counterbalanced the order of consecutive and simultaneous trials between triadic sessions. In half of the triadic sessions, the proposers started with four simultaneous trials; the other half began with four consecutive trials. At the same time, in two of the four consecutive trials, the individual on the left side participated first; in the other two consecutive trials, the individual on the right side participated first. The identity of the starting individual was also counterbalanced so that each ape started the consecutive trials first in four test sessions. Responders apparently showed no side bias. When first offers were released from the right side, responders chose the right side in 42% of trials. When first offers were released from the left side, responders chose the left side in 55% of trials.

In the other eight test sessions, chimpanzees participated in dyadic trials. Even though both proposers were present, on a given trial, only one chimpanzee had access to rewards and the wooden stick to make an offer. The other chimpanzee had no access to the rewards at all—there were no rewards on her side of the apparatus, so she could not make any offer, and they had no access to the wooden stick. Within a dyadic session, each individual made four offers, alternating their participation every two trials. Additionally, each ape started as a proposer in half of the dyadic sessions. Proposers also changed their position every two sessions. Therefore, each proposer participated eight times from the left side and eight times from the right side. From each side, they participated in four triadic and four dyadic sessions.

We baited the corresponding proposers' side in all trials with eight grapes. Apes had 20 s to send any quantity of grapes from 1 to 8 to the responder as an offer. In dyadic trials, the experimenter removed the rewards after 20 s if the apes did not send an offer to proceed with the subsequent trial. Likewise, in triadic trials, the experimenter could also remove the rewards after 20 s when both apes did not send any offer. In other words, we did not allow responders to accept any zero offer—that would have resulted in proposers obtaining eight grapes in many of those trials despite not offering any reward. Once one or two offers were made, responders had 60 s to accept one of the two offers. Responders accepted the offer within 20 s in 99.5% of trials. Once the responder had taken one of the two offers, we removed the rejected offer from one of the proposers' sides and continued with the subsequent trial. Responders could also reject offers in dyadic trials, either waiting as in the triadic scenario, or opening the access to the side of the proposer who was not participating in the task.

### Analysis plan

We performed a simulation-based power analysis to assess whether our restricted sample size was adequate to detect real differences in total responder rewards between the dyadic and triadic conditions given the two models we specify below (see the details in the ESM S1 and in https://osf.io/9m35c).

All our analyses were conducted with R-statistics (version 4.1.2). We used the brms package^45^ for our models and the ggplot2 package for our visualizations.

First, we fitted two pre-registered models to our data set, both of which used a Beta response distribution to model the proportion of maximum possible reward offered by a proposer over the course of a session (i.e., total number of grapes offered when summing across all trials divided by maximum possible total offer). In both models, the Beta distribution's mean and variance were allowed to vary between dyadic and triadic games, with both parameters also subject to random variation across triads. The mean of the distribution was also permitted to shift linearly with session number as participants gained experience, with separate slopes for the two conditions. Only random effects of triad were included, without corresponding effects of the responder or proposer(s), as the limited number of unique triads precludes the reliable separation of individual effects– in particular, no participant acted as the responder in more than one triad.

One model was applied to the complete data set, i.e., the maximum possible offer in each session is 64 (8 grapes in each of 8 trials), and so serves to contrast the dyadic and triadic conditions. The other model was applied only to the "first half" of the data, i.e., to the first four trials of each session, so that the maximum possible offer is reduced to 32. With this restriction of the data, each triadic session consists of either entirely simultaneous or entirely consecutive trials, and therefore this model includes an additional predictor variable for the mean of the beta distribution, which takes the value 0 for dyadic trials, -0.5 for simultaneous triadic trials, and 0.5 for consecutive triadic trials. Thus, the value of the corresponding "slope" parameter does not influence the fit to dyadic trial data but can capture a difference in mean between simultaneous and consecutive triadic trials.

After fitting these models, we computed 95% posterior predictive intervals for the total proportional offer in dyadic and triadic conditions (first model) and in simultaneous and consecutive triadic conditions (second model), as well as the differences between these conditions. If either of these difference intervals excluded zero, we interpreted this as solid evidence for a difference in proposer behavior between the two conditions.

Next, we fitted a series of models with a Bernoulli response distribution to model the tendency of proposers to increase their offers. The first model was applied to a variable representing, for each proposer in each session (both dyadic and triadic), whether their final offer in that session was or was not higher than their first offer in that session. This model included interacting fixed effects of session number and game type (dyadic vs. triadic). Two other models were fitted only to data from triadic sessions, specifically to variables representing, for each trial other than the first of a session by each proposer, whether their offer in that trial was (i) higher than their own offer in the preceding trial, or (ii) higher or equal than the offer accepted by the responder in the immediately preceding trial. These two trial-level models also included interacting fixed effects of session number, as well as a variable indicating whether or not the proposers' offer on the preceding trial was accepted or rejected by the responder. All models included random intercepts of proposer.

Finally, we fitted two models with Bernoulli response distributions to data from each consecutive triadic trial, specifically to a variable indicating whether or not the second proposer participating in that trial offered more than the proposer participating first. The first such model included only a fixed effect of session and random effects of the second proposer, and thus treated all consecutive trials in a given session equally. The second model stratified the consecutive trials by the value of the first offer, allowing for the possibility that second proposers were not equally likely to "outbid" a first offer of 6 or 7 as they were a first offer of 1 or 2. The first offer was included as a monotonic ordinal fixed effect; as the value of the first offer increased from 0 to 8, the model was constrained to estimate ever lower probabilities of the second proposer offering more than the first, but the decreases in probability needed not happen at a constant rate. The second model also included a fixed effect of whether the second proposer’s offer in the previous trial was accepted or rejected, and consequently this model was fitted to a slightly smaller dataset with the first trial in each session discarded. The effect of the previous trial interacted with the effect of session. See more model details in the MSF and in https://github.com/ccp-eva/competitive-altruism.git

### Pre-registration

The authors pre-registered the hypothesis, power analysis, and initial models in OSF (https://osf.io/9m35c) before the data was formally analyzed.

### Supplementary Information

Supplementary Information 1.

Supplementary Information 2.

## Data Availability

The datasets generated and/or analyzed during the current study are available in https://github.com/ccp-eva/competitive-altruism.git
