# Supplementary material for: Chimpanzees engage in competitive altruism in a triadic ultimatum game
Source: Sci Rep. 2024 Feb 9;14:3393. doi: 10.1038/s41598-024-53973-6 (PMC10858273; doi:10.1038/s41598-024-53973-6)
Supplement: Supplementary file 1 — Supplementary Information 1. [file 41598_2024_53973_MOESM1_ESM.docx]

**Chimpanzees engage in competitive altruism in a triadic Ultimatum Game**

Alejandro Sánchez-Amaro^1^, Luke Maurits^1^ & Daniel Haun^1^

^1^Department of Comparative Cultural Psychology, Max Planck Institute for Evolutionary Anthropology, Leipzig, Germany

**Supplementary materials**

Original dataset and model scripts can be found in https://github.com/ccp-eva/competitive-altruism.git

Table S1

Details of the study participants. Apes who did not participate as proposers or responders were drop-outs who were not motivated to participate or did not pass the quantity discrimination test.

| Name | Date of birth | Age at the time of test (February 2022) | Sex | Participated as proposer with | Participated as responder with |
| --- | --- | --- | --- | --- | --- |
| Sandra | 9/6/1993 | 28y 5m | Female | Azibo, Frodo, Tai |  |
| Tai | 12/8/2002 | 19y 6m | Female | Frodo, Riet (mother), Sandra | Azibo & Sandra |
| Frodo | 28/11/1993 | 28y 2m | Male | Riet, Sandra, Tai | Sandra & Tai |
| Azibo | 14/4/2015 | 6y 9m | Male | Riet, Sandra | Frodo & Tai |
| Riet | 11/11/1977 | 44y 2m | Female | Azibo, Frodo, Tai (daughter) | Frodo & Sandra (daughter) |
| Changa | 3/3/2011 | 10y 10m | Female | - | Frodo & Riet |
| Dorien | 22/10/1980 | 41y 3m | Female | - | Azibo (son) & Riet |
| Fraukje | 6/4/1976 |  | Female | - | - |
| Bambari | 8/12/2000 |  | Female | - | - |
| Kisha | 4/3/2004 |  | Female | - | - |
| Maya | 1/5/1986 |  | Female | - | - |
| Natascha | 28/3/1980 |  | Female | - | - |
| Ohini | 25/3/2016 |  | Male | - | - |
| Robert | 1/12/1975 |  | Male | - | - |
| Swela | 19/10/1995 |  | Male | - | - |

**Inter-observer reliability**

A second observer coded 18% of the dataset for inter-observer reliability. The reliability between the main coder and the second coder was excellent. Specifically, the observer coded the offer made by the proposer on the left side (Cohen's Kappa = 0.93), the offer made by the proposer on the right side (Cohen's Kappa = 0.95), and the offer accepted by the responder (Cohen's Kappa = 0.99).

**Power analysis** (pre-registered in https://osf.io/9m35c)

To simulate the interactions, we defined several strategies that might conceivably approximate the behavior of any individual chimpanzee (see details of every strategy in the pre-registration and the supplementary materials). In the simulations, all strategies are implemented probabilistically. Each chimpanzee has a "noise" parameter, sampled from a Normal distribution centered on 0.2 with a standard deviation of 0.05 (such that values are very likely to fall between 0.1 and 0.3), which dictates how closely they adhere to their strategy. Thus, multiple simulations of a game with the same assignment of strategies to participants do not yield identical outcomes.

The strategies are used for dyadic and triadic games, with an additional mechanism used for triadic games where proposals are made consecutively. Each chimpanzee has an additional "competition awareness" parameter (set to TRUE or FALSE by an independent fair coin toss for each ape) which indicates whether or not they are sensitive to the additional competitive potential of the consecutive offer scenario. Chimpanzees who are "competition aware" and make the second offer of a consecutive trial will either offer the same number of grapes as the first proposer (so as not to be "out-offered"), or will offer one grape more than the first proposer (with equal probability). Chimpanzees who are "competition unaware" and make the second offer in a consecutive trial will ignore their co-proposer's offer and act according to the same strategy they use in dyadic trials and when making the first offer in a consecutive trial.

For each separate simulation, every participant is assigned a dyadic proposer strategy, a dyadic responder strategy, a triadic responder strategy, a noise parameter (shared across all strategies), and a competition awareness parameter, which remain fixed for all games in the simulation. Chimpanzees use the same strategies and parameters for all the triads in which they participate. Due to the potential for interactions between the strategies of participating apes, simulated apes may behave quite differently across different games within the same simulation, depending on whom they are partnered with. In this way, the difference in total responder rewards between the dyadic and triadic conditions will be greater in some simulations and lesser in others, reflecting our underlying uncertainty about effect size. If our model can successfully detect a difference between dyadic and triadic conditions in the majority of simulated worlds, this provides some confidence that it is likely to succeed also in the real world.

**Strategies used for power analysis**

These strategies are not intended as direct, literal hypotheses about what happens inside the mind of real-world chimpanzees. More sophisticated strategies are naturally possible (and indeed likely), as is the prospect of participants changing strategies over time. Instead, these strategies are intended as principled, non-arbitrary tools to provide "ballpark" estimates of many quantities necessary for power analysis, such as the possible differences in total proposer offer between dyadic and triadic conditions or the amount of variation within conditions across triads.

*Dyadic responder strategies*

Rational Dyadic Responder: Accepts any non-zero offer – "anything is better than nothing". This strategy is based on previous results with great apes (e.g., Jensen et al., 2007).

Fairness Dyadic Responder: Accepts any offer where they get at least as much reward as the proposer – "I should forgo rewards to punish the greedy".

No Decrease Dyadic Responder: Rejects initial offers of zero and rejects any subsequent offer which is not at least as high as the highest previous offer in the same game – "I know you gave me n grapes once before, so I will never accept less than n again".

*Triadic responder strategies*

Rational Triadic Responder: Accepts the greatest of the two offers, choosing randomly if both offers are equal – "the more for me, the better".

Fairness Triadic Responder: Does not distinguish between fair offers (i.e., those where the responder gets at least as many grapes as the proposer), choosing amongst such offers randomly. Acts as a Rational Dyadic Responder if unfair offers are involved – "I am more concerned with not being taken advantage of by a greedy proposer than I am with maximizing my rewards".

Maximum Generosity Responder: Accepts the offer of whichever proposer has offered the highest cumulative total of grapes across all rounds of the game so far, regardless of the current offers - "Your last-minute generosity does not make up for consistent early stinginess".

*Common proposer strategies (available for use in both dyadic and triadic games)*

Rational Proposer: Always offers a single grape, secure that a correspondingly Rational Responder will always accept this, leaving a large reward for the proposer.

Reluctantly Increasing Proposer: Each such proposer has a threshold number n of rejections (n=1 with p=0.8, n=2 with p=0.15, n=3 with p=0.05). They repeat their previous offer until this has been rejected (dyadic condition) or the other proposer's offer chosen instead (triadic condition) a total of n times in a row, after which they increase their offer by one grape. The initial offer is a low offer of 1, 2 or 3 grapes (with the same probability distribution as n above). This strategy is based on the famous win-stays, lose-shifts game theory strategy by Nowak and Sigmund (Nowak & Sigmund, 1993).

*Triadic-only proposer strategies*

Repeat Last Proposer: Makes a low initial offer as per the Reluctantly Increasing Proposer. For all subsequent rounds, offers the same amount that the responder accepted on the previous round – "You accepted these many grapes last time; you should accept them again".

We performed a total of 500 simulations, each involving seven simulated apes organized into seven triads according to constraints inferred from the performance of actual apes in pre-testing phases, i.e. that two apes would be unable to play the role of proposer. The seven triads were carefully structured so as to minimise variation in the degree of similarity between pairs of triads. There are 21 distinct pairs of triads. 11 of these pairs have a single ape in common, while two pairs have no overlap at all. The remaining 8 pairs have two apes in common – in three of these cases, both apes play different roles in the two triads.

The model described below was applied to all 500 simulated datasets (the experience variable was omitted, as the strategies used in our simulations were incapable of producing genuine effects of experience). In 469 of these analyses, a difference between the dyadic and triadic games was inferred with high confidence (as per the description below), giving an estimated power of 0.94, well above the conventional threshold of 0.80.

Note that a detectable difference in proposer’s offers between the dyadic and triadic conditions does not necessarily imply an understanding of the proposers that they are participating in a competitive scenario. For example, two proposers who pay no attention to each other’s offers and act simply as Reluctantly Increasing Proposers may, when paired with a rational responder, end up increasing their offers, even if both make equal initial offers. If the randomly choosing responder happens to accept one proposer’s offers sufficiently many times in a row to cause the other proposer to increase their offer, the responder will “switch sides”, eventually causing the other proposer to increase their offer, at which point both proposers are making equal offers and the initial dynamic may repeat. Therefore, we perform a second power analysis focused on detecting a difference between consecutive and simultaneous triadic trials, based only on the first 4 rounds of each sessions. Such an effect can only be reasonably explained via competitive reasoning on the part of participants. Again we performed 500 simulations, and an effect of consecutive vs simultaneous trial structure was found in only 259 analyses, giving an estimated power of 0.52.

This considerable decrease in power is expected in light of effectively halving the amount of data by focusing only on the first 4 rounds. However, the power also depends upon how common it is for participants to understand the competitive nature of the task. In the simulations above, this “competitive awareness” parameter was set for each participant by flipping a fairly weighted coin, i.e. on average only half the participants in the simulated population are competitively aware, and therefore triads in which both proposers are consciously trying to out-compete the other are quite rare. As we increase the probability of simulated chimps being competitively aware, the power increases too. At competitive awareness probabilities of 0.65, 0.75 and 0.85, we estimated powers for the second model of of 0.67, 0.75 and 0.81, respectively.

**Descriptive plots**

Figure S1


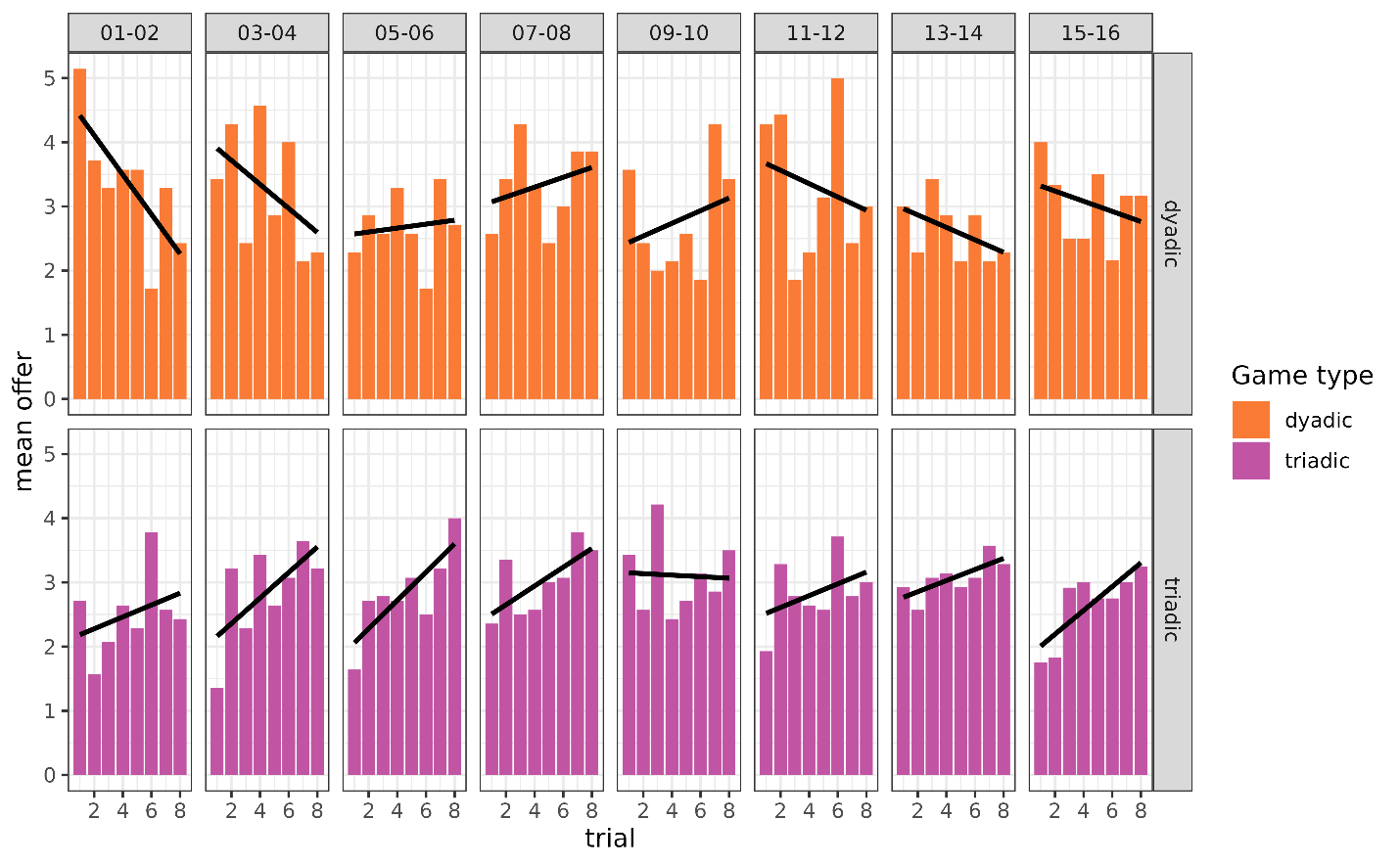


Figure S1: Mean offer trial-by-trial for sessions 1 to 16 in dyadic and triadic conditions.

Figure S2


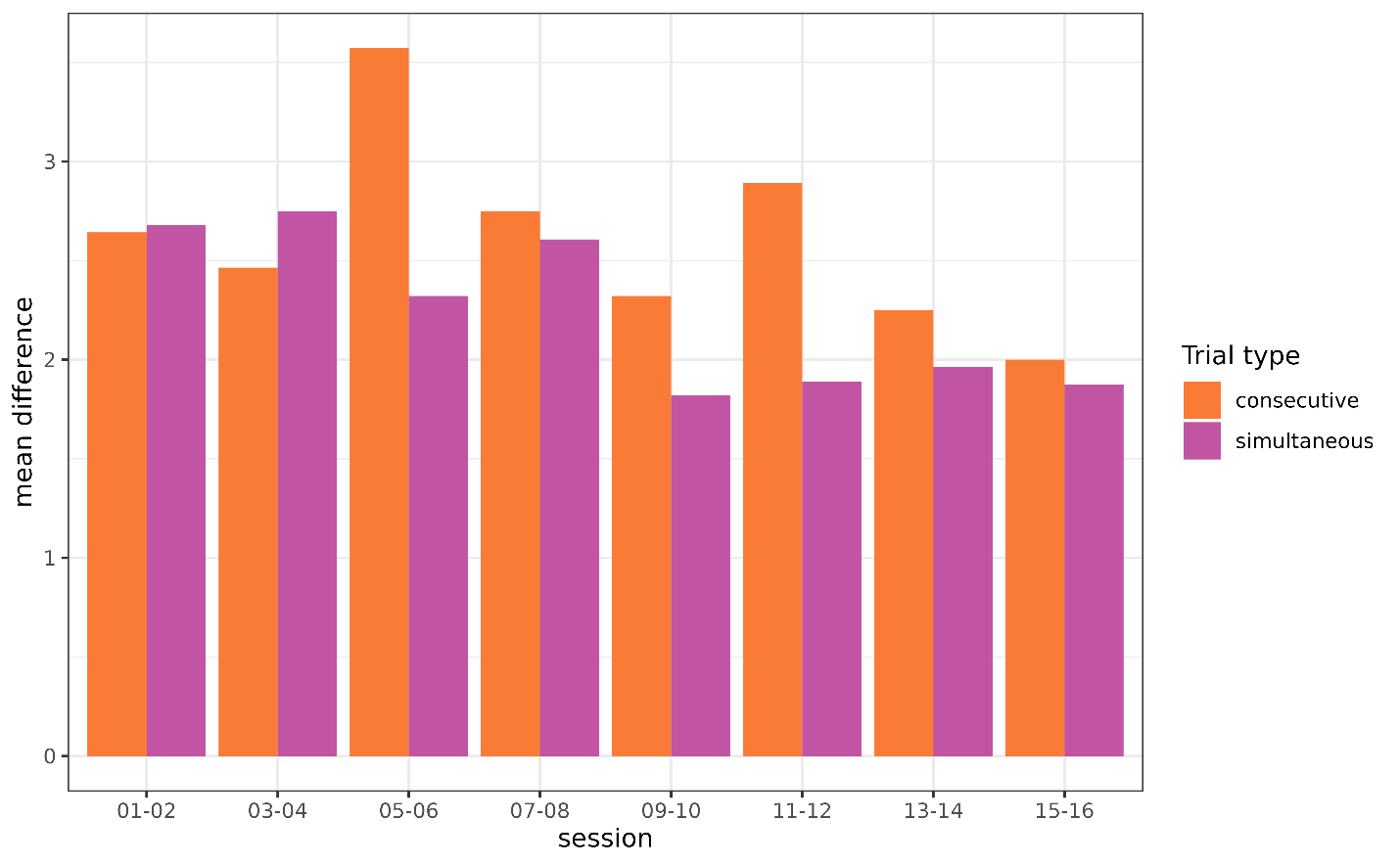


Figure S2: Mean absolute difference between both offers in consecutive and in simultaneous triadic trials.

Figure S3


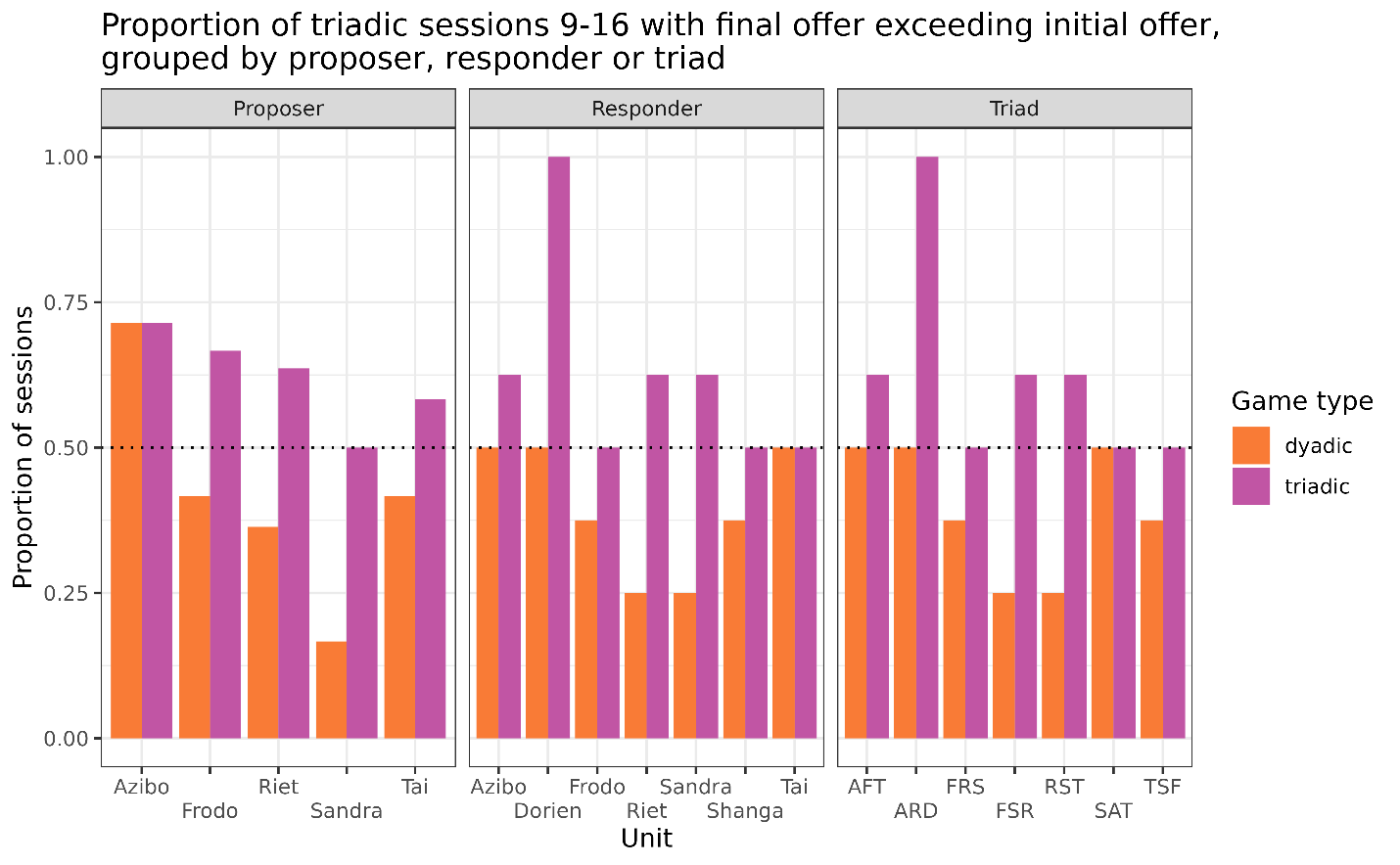


Figure S3: Proportion of sessions 9-16 with final offer exceeding initial offer grouped by proposer, responder, and triad. In orange we depict the dyadic condition and in purple the triadic condition.

Figure S4


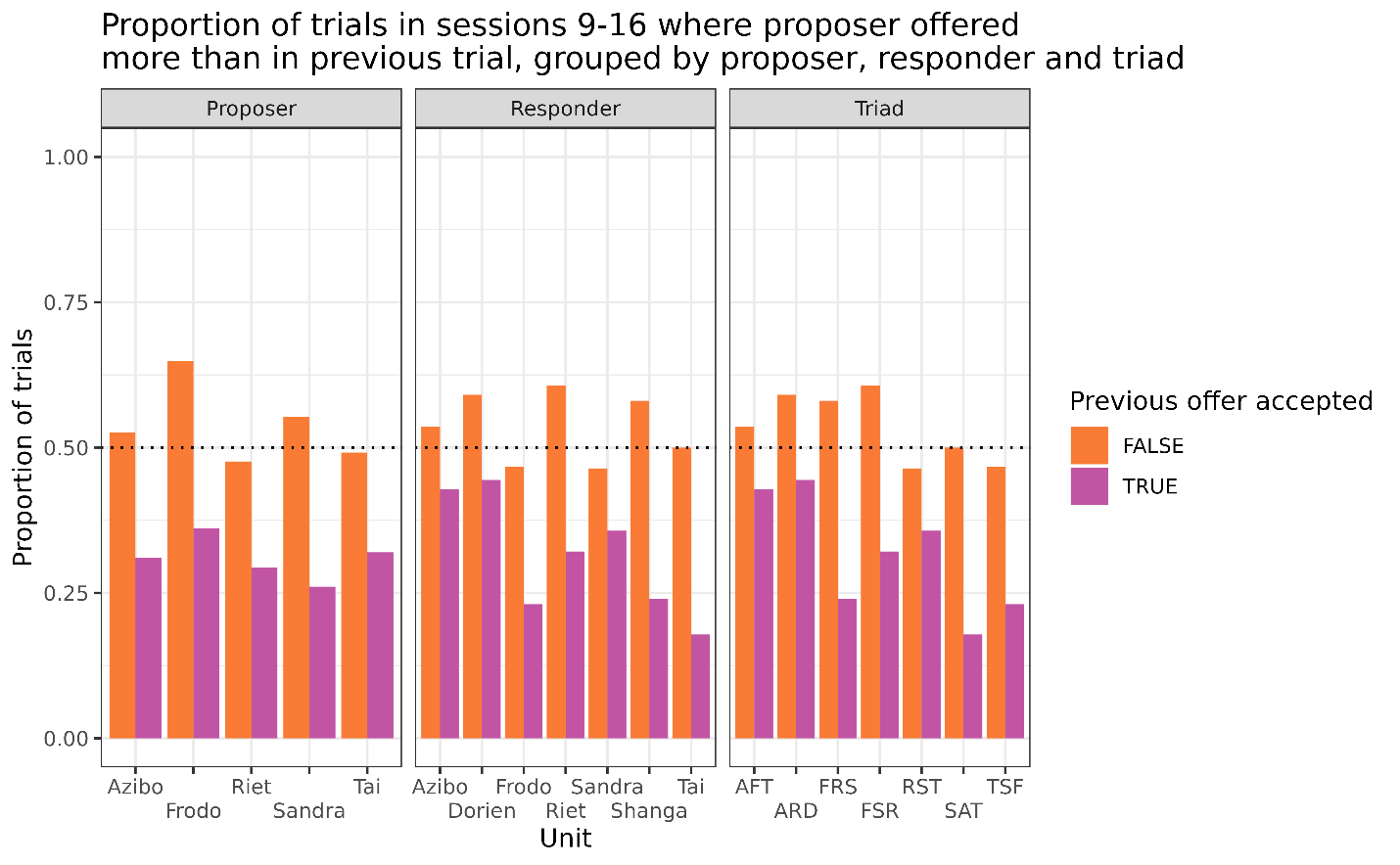
Figure S4: Proportion of trials in sessions 9-16 where proposes offered more than in previous trials grouped by proposer, responder and triad. In orange we depict instances in which the previous offer had been rejected and in purple instances in which the previous offer had been accepted.

Figure S5


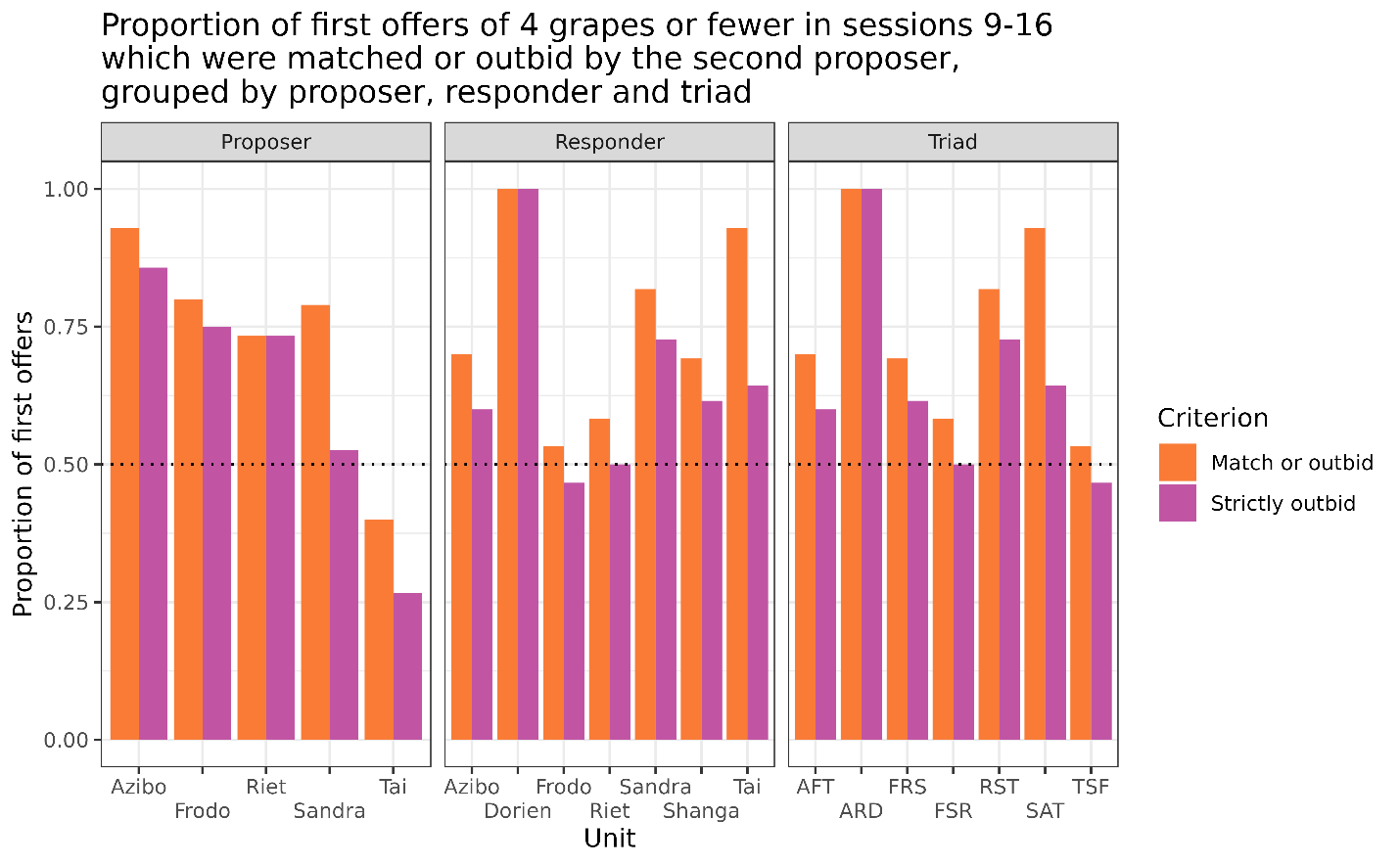


Figure S5: Proportion of first offers of 0 to 4 grapes during sessions 9-16 which were matched or outbid by the second proposer, grouped by proposer, responder and triad. In orange we plot whether the offer as matched our outbid. In purple we plot whether the offer was strictly outbid.
