# Supplementary material for: Chimpanzees engage in competitive altruism in a triadic ultimatum game
Source: Sci Rep. 2024 Feb 9;14:3393. doi: 10.1038/s41598-024-53973-6 (PMC10858273; doi:10.1038/s41598-024-53973-6)
Supplement: Supplementary file 2 — Supplementary Information 2. [file 41598_2024_53973_MOESM2_ESM.pdf]

Chimpanzees engage in competitive altruism in a triadic Ultimatum Game

Model summaries

Luke Maurits <luke_maurits@eva.mpg.de>

2024-01-12

NB: Throughout, predictions for the "8.5th" session refer to those obtained with the centered session number predictor set to zero. Because the total number of sessions is even, this does not directly correspond to any particular actual experimetnal session. Nevertheless, it faithly represents expected performance "half way through" any inferred learning effects.

1 Pre-registered models of total proportional offer

1.1 M1: Proportional total offer, dyadic vs triadic

1.1.1 Posterior predictive distribution for total proportional offers in "8.5th" session

	Posterior mean	95% HPD lower	95% HPD upper
Dyadic	0.37	0.01	0.90
Triadic	0.31	0.02	0.75

1.1.2 Posterior predictive distribution for difference in total proportional offers in "8.5th" session

Posterior mean	95% HPD lower	95% HPD upper
-0.06	-0.66	0.53

1.2 M2: Proportional total offer, consecutive vs simultaneous

1.2.1 Posterior predictive distribution for total proportional offers in "8.5th" triadic session

	Posterior mean	95% HPD lower	95% HPD upper
Simultaneous	0.25	0.00	0.76
Consecutive	0.31	0.01	0.83

Posterior probability that mean consecutive offer is higher than mean simulteanous offer:

```
## [1] 0.82375
```

Posterior predictive distribution for difference in offer between conditions:

Posterior mean	95% HPD lower	95% HPD upper
0.07	-0.56	0.7

2 Models of tendency for final offfers to exceed initial offers within sessions

2.0.1 Marginalised over session probabilities of increasing within a session

	Posterior mean	95% HPD lower	95% HPD upper
Dyadic	0.37	0.23	0.53
Triadic	0.57	0.39	0.73

2.0.2 Per-session probabilities

2.0.2.1 Dyadic

Session	Posterior mean	95% HPD lower	95% HPD upper
1	0.34	0.19	0.52
2	0.34	0.20	0.51
3	0.35	0.21	0.51
4	0.35	0.23	0.50
5	0.36	0.24	0.50
6	0.36	0.24	0.50
7	0.37	0.25	0.50
8	0.37	0.26	0.50
9	0.38	0.26	0.51
10	0.38	0.26	0.51
11	0.39	0.26	0.52
12	0.39	0.26	0.53
13	0.40	0.26	0.55
14	0.40	0.25	0.56
15	0.41	0.25	0.58
16	0.41	0.24	0.59

2.0.2.2 Triadic

Session	Posterior mean	95% HPD lower	95% HPD upper
1	0.52	0.33	0.70
2	0.53	0.35	0.70
3	0.53	0.36	0.70
4	0.54	0.38	0.69
5	0.55	0.39	0.69
6	0.56	0.40	0.69
7	0.56	0.41	0.70
8	0.57	0.42	0.70
9	0.58	0.43	0.71
10	0.58	0.43	0.72
11	0.59	0.44	0.73
12	0.60	0.44	0.74
13	0.61	0.44	0.75
14	0.61	0.44	0.77
15	0.62	0.44	0.78
16	0.63	0.43	0.79

2.0.2.3 Per session contrast in probabilities (triadic - dyadic)

Session	Posterior mean	95% HPD lower	95% HPD upper
1	0.18	-0.04	0.37
2	0.18	-0.03	0.37
3	0.18	-0.01	0.36
4	0.19	0.00	0.35
5	0.19	0.01	0.35
6	0.19	0.01	0.34
7	0.20	0.02	0.34
8	0.20	0.02	0.34
9	0.20	0.02	0.34
10	0.20	0.02	0.35
11	0.20	0.02	0.35
12	0.21	0.02	0.36
13	0.21	0.01	0.37
14	0.21	0.00	0.38
15	0.21	0.00	0.39
16	0.21	-0.01	0.40

2.0.3 Per-triad contrast in probabilities (traidic - dyadic) in "8.5th" session

Triad	Posterior mean	Post. prob. of positive contrast
AFT	0.17	0.92
ARD	0.28	1.00
FRS	0.23	0.99
FSR	0.20	0.96
RST	0.20	0.96
SAT	0.26	0.99
TSF	0.17	0.92

3 Models of tendency to increase offers from trial to trial

3.1 Relative to self

3.1.1 Marginalised over sessions

	Posterior mean	95% HPD lower	95% HPD upper
Previous offer accepted	0.29	0.19	0.42
Previous offer rejected	0.51	0.36	0.64

3.1.2 Accepted vs rejected contrast

Posterior mean	95% HPD lower	95% HPD upper
-0.21	-0.33	-0.06

3.1.3 Per-proposer and responder accepted vs rejected contrasts in "8.5th" session

Proposer	Responder	Posterior mean	Post. prob. of negative contrast
Azibo	Dorien	-0.23	1
Azibo	Tai	-0.22	1
Frodo	Azibo	-0.23	1
Frodo	Riet	-0.23	1
Frodo	Shanga	-0.23	1
Riet	Dorien	-0.20	1
Riet	Sandra	-0.20	1
Riet	Shanga	-0.20	1
Sandra	Frodo	-0.25	1
Sandra	Riet	-0.26	1
Sandra	Tai	-0.26	1
Tai	Azibo	-0.23	1
Tai	Frodo	-0.22	1
Tai	Sandra	-0.23	1

3.1.4 Separating consecutive and simultaneous trials

3.1.4.1 Posterior probability of higher increasing probability in consecutive trials than simultaneous

```
## [1] 0.793
```

3.1.4.2 Posterior probability of increasing offers for different trial types

Previous offer	Trial type	Posterior mean	95% HPD lower	95% HPD upper
Accepted	Triadic consecutive	0.30	0.19	0.43
Accepted	Triadic simultaneous	0.28	0.17	0.42
Rejected	Triadic consecutive	0.53	0.39	0.67
Rejected	Triadic simultaneous	0.48	0.33	0.63

3.2 Relative to previous trial winner

NB: here we consider increasing over *or* matching the previous winner.

3.2.1 Marginalised over sessions

	Posterior mean	95% HPD lower	95% HPD upper
Previous offer accepted	0.45	0.28	0.63
Previous offer rejected	0.36	0.21	0.54

3.2.2 Accepted vs rejected contrast

Posterior mean	95% HPD lower	95% HPD upper
0.09	-0.03	0.2

4 Models of tendency of second proposers to outbid first proposers

4.1 Without accounting for value of first offer

4.1.1 Posterior probability that second proposer becomes more likely to outbid first proposer as sessions increase

```
## [1] 0.76225
```

4.1.2 Probability that second proposer offers more than first proposer in consecutive trials, marginalising over sessions

Posterior mean	95% HPD lower	95% HPD upper
0.45	0.25	0.67

4.1.3 Per session probabilities that second proposer offers more than first proposer in consecutive trials

Session	Posterior mean	95% HPD lower	95% HPD upper
1	0.41	0.22	0.63
2	0.41	0.23	0.63
3	0.42	0.24	0.63
4	0.42	0.24	0.62
5	0.43	0.25	0.62
6	0.44	0.25	0.63
7	0.44	0.26	0.63
8	0.45	0.26	0.64
9	0.45	0.26	0.65
10	0.46	0.26	0.65
11	0.46	0.26	0.66
12	0.47	0.26	0.67
13	0.47	0.26	0.69
14	0.48	0.26	0.70
15	0.49	0.25	0.70
16	0.49	0.25	0.72

4.1.4 Per second proposer and responder probabilities that second proposer offers more than first proposer in consecutive trials for "8.5th" session

Second proposer	Responder	Posterior mean	95% HPD lower	95% HPD upper
Azibo	Dorien	0.62	0.43	0.81
Azibo	Tai	0.59	0.39	0.78
Frodo	Azibo	0.53	0.38	0.70
Frodo	Riet	0.50	0.34	0.65
Frodo	Shanga	0.53	0.38	0.69
Riet	Dorien	0.45	0.28	0.61
Riet	Sandra	0.49	0.34	0.67
Riet	Shanga	0.45	0.29	0.60
Sandra	Frodo	0.41	0.26	0.57
Sandra	Riet	0.40	0.26	0.56
Sandra	Tai	0.40	0.25	0.55
Tai	Azibo	0.30	0.16	0.47
Tai	Frodo	0.28	0.13	0.44
Tai	Sandra	0.34	0.19	0.53

4.2 Stratifying by first offer

4.2.1 Estimated offering preferences

A probability distribution reflecting our estimate of proposer's preferred offers in the absence of competition was calculated on the basis of first offers in consecutive trials. A distribution was calculated separately for sessions 1 through 16 as follows. In each session and for each possible offer, the proportion of observed offers in the 1st, 2nd, 3rd,. . . , 8th trial equal to that possible offer was computed. The session probability for that offer was set to the weighted mean of these per-trial probabilities, with each trial receiving half the weight of the previous trial, i.e. 2nd trial data has half the weight of 1st trial data, 3rd trial data has half the weight of 2nd trial data, and so on. Under this scheme, approximately 95% of the contribution to the estimate comes from the first half of session. The 16 session distributions are then combined into a weighted mean where each session receives 3/4 the weight of the previous session, i.e. influence decays across sessions less steeply than across trials, since sessions are separated by a much longer time. The table below shows both the final offering probabilities and the baseline outbidding probabilities estimated on the basis of 10,000 simulated trials of comparing "first" and "second" offers drawn from the preferred offer distribution.

Offer	Preference probability	Baseline outbiding probability
0	0.23	0.77
1	0.15	0.62
2	0.17	0.45
3	0.19	0.26
4	0.10	0.17
5	0.08	0.08
6	0.03	0.08
7	0.04	0.03
8	0.02	0.00

4.2.2 Posterior probability that second proposer becomes more likely to outbid first proposer as sessions increase

4.2.2.1 With previous offer rejected:

```
## [1] 0.7164
```

4.2.2.2 With previous offer accepted:

```
## [1] 0.4464
```

4.2.3 Outbidding probabilities in first session, stratified by first offer

First Offer	Baseline	Previously accepted	Posterior mean	95% HPD lower	95% HPD upper	PP rate above chance	PP rate above baseline
0	0.77	FALSE	0.82	0.54	0.96	0.98	0.76
1	0.62	FALSE	0.71	0.35	0.92	0.91	0.76
2	0.45	FALSE	0.52	0.17	0.84	0.54	0.65
3	0.26	FALSE	0.46	0.13	0.82	0.39	0.87
4	0.17	FALSE	0.33	0.05	0.77	0.17	0.79
5	0.08	FALSE	0.13	0.01	0.65	0.04	0.52
6	0.08	FALSE	0.08	0.00	0.56	0.03	0.25
7	0.03	FALSE	0.06	0.00	0.51	0.03	0.35
8	0.00	FALSE	0.04	0.00	0.43	0.02	1.00
0	0.77	TRUE	0.82	0.53	0.96	0.98	0.75
1	0.62	TRUE	0.70	0.35	0.92	0.90	0.74
2	0.45	TRUE	0.51	0.16	0.84	0.52	0.63
3	0.26	TRUE	0.45	0.12	0.82	0.38	0.84
4	0.17	TRUE	0.32	0.05	0.76	0.17	0.77
5	0.08	TRUE	0.13	0.01	0.64	0.04	0.51
6	0.08	TRUE	0.08	0.00	0.56	0.03	0.25
7	0.03	TRUE	0.06	0.00	0.52	0.03	0.34
8	0.00	TRUE	0.04	0.00	0.41	0.02	1.00

4.2.4 Outbidding probabilities in final session, stratified by first offer

First Offer	Baseline	Previously accepted	Posterior mean	95% HPD lower	95% HPD upper	PP rate above chance	PP rate above baseline
0	0.77	FALSE	0.88	0.66	0.98	1.00	0.92
1	0.62	FALSE	0.80	0.47	0.96	0.97	0.91
2	0.45	FALSE	0.63	0.26	0.90	0.79	0.86
3	0.26	FALSE	0.57	0.20	0.89	0.67	0.95
4	0.17	FALSE	0.44	0.09	0.85	0.36	0.91
5	0.08	FALSE	0.19	0.01	0.77	0.08	0.68
6	0.08	FALSE	0.12	0.00	0.69	0.04	0.39
7	0.03	FALSE	0.09	0.00	0.64	0.04	0.49
8	0.00	FALSE	0.06	0.00	0.57	0.03	1.00
0	0.77	TRUE	0.83	0.57	0.96	0.99	0.79
1	0.62	TRUE	0.72	0.37	0.93	0.92	0.78
2	0.45	TRUE	0.53	0.18	0.85	0.58	0.68
3	0.26	TRUE	0.47	0.14	0.83	0.43	0.87
4	0.17	TRUE	0.34	0.06	0.78	0.19	0.81
5	0.08	TRUE	0.14	0.01	0.66	0.05	0.54
6	0.08	TRUE	0.09	0.00	0.57	0.03	0.27
7	0.03	TRUE	0.07	0.00	0.52	0.03	0.37
8	0.00	TRUE	0.04	0.00	0.44	0.02	1.00

4.2.5 Per second proposer and per responder posterior predictions on outbidding initial offers of 1, 2, 3 or 4 in final session

4.2.5.1 Previous offer accepted

4.2.5.1.1 Posterior probabilities that outbidding rates are higher than chance

Second proposer	Responder	1	2	3	4
Azibo	Dorien	1.00	0.99	0.97	0.89
Azibo	Tai	0.99	0.94	0.89	0.69
Frodo	Azibo	0.89	0.53	0.39	0.16
Frodo	Riet	0.94	0.66	0.52	0.22
Frodo	Shanga	0.95	0.70	0.57	0.30
Riet	Dorien	0.98	0.86	0.78	0.53
Riet	Sandra	0.98	0.84	0.74	0.43
Riet	Shanga	0.97	0.78	0.67	0.35
Sandra	Frodo	0.54	0.12	0.06	0.01
Sandra	Riet	0.87	0.47	0.31	0.08
Sandra	Tai	0.84	0.40	0.25	0.06
Tai	Azibo	0.75	0.23	0.12	0.02
Tai	Frodo	0.45	0.07	0.03	0.01
Tai	Sandra	0.87	0.46	0.31	0.10

4.2.5.1.2 Posterior probabilities that outbidding rates are above the estimated baseline

Second proposer	Responder	1	2	3	4
Azibo	Dorien	1.00	0.99	0.98	0.92
Azibo	Tai	0.99	0.94	0.91	0.80
Frodo	Azibo	0.91	0.71	0.62	0.41
Frodo	Riet	0.94	0.77	0.70	0.50
Frodo	Shanga	0.96	0.79	0.73	0.53
Riet	Dorien	0.98	0.90	0.85	0.70
Riet	Sandra	0.98	0.88	0.82	0.65
Riet	Shanga	0.97	0.84	0.78	0.59
Sandra	Frodo	0.70	0.37	0.27	0.10
Sandra	Riet	0.91	0.68	0.57	0.32
Sandra	Tai	0.88	0.62	0.51	0.28
Tai	Azibo	0.83	0.49	0.38	0.16
Tai	Frodo	0.63	0.27	0.19	0.07
Tai	Sandra	0.91	0.65	0.54	0.30

4.2.5.2 Previous offer rejected

4.2.5.2.1 Posterior probabilities that outbidding rates are higher than chance

Second proposer	Responder	1	2	3	4
Azibo	Dorien	0.99	0.96	0.94	0.84
Azibo	Tai	0.98	0.93	0.88	0.68
Frodo	Azibo	0.96	0.77	0.68	0.43
Frodo	Riet	0.98	0.86	0.79	0.53
Frodo	Shanga	0.98	0.88	0.81	0.57
Riet	Dorien	0.99	0.95	0.91	0.72
Riet	Sandra	0.99	0.94	0.89	0.65
Riet	Shanga	0.99	0.91	0.84	0.58
Sandra	Frodo	0.89	0.51	0.36	0.12
Sandra	Riet	0.99	0.90	0.80	0.43
Sandra	Tai	0.99	0.86	0.74	0.36
Tai	Azibo	0.87	0.47	0.30	0.08
Tai	Frodo	0.66	0.17	0.08	0.01
Tai	Sandra	0.94	0.70	0.54	0.21

4.2.5.2.2 Posterior probabilities that outbidding rates are above the estimated baseline

Second proposer	Responder	1	2	3	4
Azibo	Dorien	0.99	0.97	0.95	0.88
Azibo	Tai	0.98	0.94	0.91	0.79
Frodo	Azibo	0.96	0.85	0.79	0.62
Frodo	Riet	0.98	0.90	0.86	0.69
Frodo	Shanga	0.98	0.91	0.87	0.72
Riet	Dorien	0.99	0.95	0.92	0.81
Riet	Sandra	0.99	0.95	0.91	0.77
Riet	Shanga	0.99	0.92	0.88	0.73
Sandra	Frodo	0.92	0.69	0.59	0.35
Sandra	Riet	0.99	0.92	0.85	0.65
Sandra	Tai	0.98	0.90	0.82	0.59
Tai	Azibo	0.91	0.66	0.55	0.30
Tai	Frodo	0.78	0.42	0.31	0.13
Tai	Sandra	0.95	0.80	0.70	0.44
